# The mungbean *VrP* locus encoding MYB90, an R2R3-type MYB protein, regulates anthocyanin biosynthesis

**DOI:** 10.3389/fpls.2022.895634

**Published:** 2022-07-22

**Authors:** Yun Lin, Kularb Laosatit, Jinyang Liu, Jingbing Chen, Xingxing Yuan, Prakit Somta, Xin Chen

**Affiliations:** ^1^Jiangsu Key Laboratory for Horticultural Crop Genetic Improvement, Institute of Industrial Crops, Jiangsu Academy of Agricultural Sciences, Nanjing, China; ^2^Department of Agronomy, Faculty of Agriculture at Kamphaeng Saen, Kasetsart University, Kamphaeng Saen, Thailand

**Keywords:** mungbean, *Vigna radiata*, anthocyanins, MYB90, hypocotyl color

## Abstract

Anthocyanins are water-soluble pigments present in several tissues/parts of plants. The pigments provide color and are wildly known for health benefits for human, insect attraction for plant pollination, and stress resistance in plants. Anthocyanin content variations in mungbean [*Vigna radiata* (L.) Wilczek] were first noticed a long time ago, but the genetic mechanism controlling the anthocyanins in mungbean remains unknown. An F_2_ population derived from the cross between purple-hypocotyl (V2709) and green-hypocotyl (Sulv1) mungbeans was used to map the *VrP* locus controlling purple hypocotyl. The *VrP* locus was mapped to a 78.9-kb region on chromosome 4. Sequence comparison and gene expression analysis identified an R2R3-MYB gene *VrMYB90* as the candidate gene for the *VrP* locus. Haplotype analysis using 124 mungbean accessions suggested that 10 single nucleotide polymorphisms (SNPs) in exon 3 may lead to an abolished expression of *VrMYB90* and an absence of anthocyanin accumulation in the hypocotyl of Sulv1 and KPS2. The overexpression of *VrMYB90* in mungbean hairy root, tobacco leaf, and *Arabidopsis* resulted in anthocyanin accumulation (purple color). Gene expression analysis demonstrated that *VrMYB90* regulated anthocyanin accumulation in the hypocotyl, stem, petiole, and flowers, and the expression was sensitive to light. VrMYB90 protein may upregulate *VrDFR* encoding dihydroflavonol 4-reductase at the late biosynthesis step of anthocyanins in mungbeans. These results suggest that *VrMYB90* is the dominator in the spatiotemporal regulation of anthocyanin biosynthesis. Our results provide insight into the biosynthesis mechanism of anthocyanin and a theoretical basis for breeding mungbeans.

## Introduction

Anthocyanins, a flavonoid, are natural water-soluble pigments abundant in plants that endow tissues and organs with distinctive colors (Glover and Martin, 2012). The color of flowers, leaves, seeds, and fruits plays an important role in the specificity of pollinators and predators (Bradshaw and Schemske, 2003; Carlson and Holsinger, 2013; Esfeld et al., 2018). Due to their high antioxidant activity, anthocyanins can enhance the tolerance and resistance of plants to biotic and abiotic stresses, such as pathogens, ultraviolet B radiation, chilling, drought, and salinity by scavenging excessive reactive oxygen species (ROS; Nakabayashi et al., 2014; Asem et al., 2015; Matus, 2016; Li et al., 2017; Wang et al., 2017; Xu and Rothstein, 2018; Bai et al., 2020; Sicilia et al., 2020). Additionally, anthocyanins show tremendous potential benefits for human and livestock health by reducing adverse health risks, especially from chronic diseases and aging (Blesso, 2019; Tena et al., 2020). These biochemical characteristics mean that anthocyanins play a significant role during crop breeding (Allan and Espley, 2018; Chaves-Silva et al., 2018) and plant evolution (Wessinger and Rausher, 2012; Davies et al., 2020; Saigo et al., 2020; Berardi et al., 2021).

Anthocyanins are secondary metabolism products produced by the flavonoid branch of the phenylpropanoid pathway. These compounds are synthesized in the cytosol and transported into the vacuole for storage. The biosynthesis of anthocyanins and regulatory pathways is well elucidated in model plant species, including *Arabidopsis thaliana* (L.) Heynh., *Petunia hybrida, Antirrhinum majus* L., and *Medicago truncatula* Gaertn. (Carletti et al., 2013; Albert et al., 2014; Bulgakov et al., 2017). In recent years, several key genes involved in the mechanical steps of the anthocyanin biosynthesis pathway have been identified in several horticultural plants (Liu et al., 2017, 2020; Yao et al., 2017; Wang et al., 2019; Zhong et al., 2020). Collectively, anthocyanin biosynthesis genes are simply divided into early biosynthesis genes (EBGs) and late biosynthesis genes (LBGs). The EBGs encoded different enzymes, including chalcone synthase (CHS), chalcone isomerase (CHI), flavanone 3-hydroxylase, and 3′-hydroxylase (F3H and F3′H), which catalyzes the production of dihydroflavonols, dihydroquercetin, or dihydromyricetin as the precursor substrates for late anthocyanin synthesis, while the LBGs mainly encoded enzymes of dihydroflavonol 4-reductase (DFR), leucoanthocyanidin dioxygenase/anthocyanidin synthase (LDOX/ANS), and UDP-glucose: flavonoid 3-*O*-glucosyltransferase (UFGT) that converted flavononols to anthocyanin (Jaakola, 2013; Chaves-Silva et al., 2018). Anthocyanins are transported and sequestrated into the vacuole as coalescences through multidrug and toxic extrusion (MATE) or ATP-binding cassette (ABC) transporters (Zhao and Dixon, 2010; Zhao et al., 2011).

In plants, the expression of anthocyanin biosynthetic genes is transcriptionally coordinated by an MYB-bHLH-WD40 (MBW) ternary complex. An R2R3-MYB transcription factor (TF) is the core activator that upregulates the expression of EBGs and LBGs by binding to the *cis*-elements on their promoters (Allan and Espley, 2018). In strawberry (*Fragaria* spp.), an R2R3 MYB member, *MYB10* is considered to be the major gene stimulating the biosynthetic genes of anthocyanins (Medina-Puche et al., 2014). In purple carrot (*Daucus carota* L.), a root-specifically expressed R2R3-MYB gene, *DcMYB113*, activates several structural genes and conditions anthocyanin biosynthesis in taproots (Xu et al., 2020). In wild European bilberry (*Vaccinium myrtillus* L.), three MYB genes, *VmMYBA1, VmMYBPA1.1*, and *VmMYBPA2.2*, were characterized as co-regulating anthocyanin accumulation in ripening fruits (Karppinen et al., 2021). In grape (*Vitis vinifera* L.), the *VvMYBA1* gene controls the peel color in the purple grape variety “Cabernet” (Kobayashi et al., 2004). Kobayashi et al. (2004) demonstrated that when a transposon was inserted into the promoter of *VvMYBA1*, it inhibited the expression of this gene and resulted in a white peel, forming the white grape variety “Chardonnay.” However, when the inserted site caused a rearrangement of the promoter sequence in the variety “Ruby Okuyama,” the expression of *Vvmyba1* was partially restored and led to the pink-peel phenotype.

bHLH TFs are co-activators of MYBs that enhance the transcriptional ability of MYB TFs. The co-expression of *bHLH* and *MYB* genes can significantly determine anthocyanin production in transgenic plants (Wang et al., 2019). Some studies propose that bHLHs can bind to the promoters of some key enzyme genes (Zhu et al., 2015; Li et al., 2020; Tao et al., 2020). WD40 was first discovered to be involved in anthocyanin biosynthesis in *Arabidopsis* (Walker et al., 1999), and its homologs have been characterized in many other species. It works as a scaffold in the MBW heterotrimer, and the interaction of WD40 with bHLH can enhance the transcriptional activation ability of MYB TFs (Xu et al., 2013). Repression or mutations in either of the regulatory genes will lead to a lack of pigmentation (Kobayashi et al., 2004; Schwinn et al., 2006; Gillman et al., 2011; Zhi et al., 2020; Meng et al., 2021; Qin et al., 2021; Yang et al., 2021).

Mungbean (*Vigna radiata* L.) is an economic and versatile legume crop of Asia and is now gaining popularity in Australia, Africa, and United States. Dry seeds of mungbean are consumed in various ways and can be processed into sprouts, noodles, protein concentrates and isolates, and starches (Nair and Schreinemachers, 2020). There are two color types of the hypocotyl in mungbean, namely, green and purple. The hypocotyl color is often used as a marker by farmers to identify cultivar purity and plant breeders to identify hybrids and selection. In addition, the hypocotyl color is also an important criterion in mungbean breeding. For example, in Thailand, breeders always select mungbean plants/lines with green hypocotyls as the trait affects the quality (color) of the mungbean sprouts. Genetic studies clearly revealed that the trait is controlled by a single locus and the purple hypocotyl is dominant (Sen and Ghosh, 1959; Swindell and Poehlman, 1978; Pandey et al., 1989). Sen and Ghosh (1959) designated locus “*P*” for purple-colored hypocotyl. However, the physical location and molecular basis of the locus *P* have not yet been investigated. Considering the nutritive values of mungbean and the benefits of anthocyanins, it is worthwhile to improve our understanding of the regulatory mechanism of anthocyanin biosynthesis in mungbean.

In this study, we reported map-based cloning and molecular characterization of the “*VrP*” locus controlling the purple hypocotyl color in mungbean. We showed that an R2R3-MYB gene, designated *“VrMYB90*,” is the candidate gene at the *VrP* locus. The transcript of *VrMYB90* is abolished in green-hypocotyl mungbean accessions, leading to the loss of purple pigmentation. The expression level of *VrMYB90* was tightly related to the anthocyanin content and was sensitive to light. Sequencing of *VrMYB90* in 124 mungbean accessions revealed that 10 single nucleotide polymorphisms (SNPs) in exon 3 of this gene were associated with green hypocotyl color. Results from this study provide insight into the anthocyanin biosynthesis mechanism and a theoretical basis for the breeding of mungbean.

## Materials and methods

### Mapping population

An F_2_ population comprising 849 individuals derived from a cross between “V2709” (purple hypocotyl) and “Sulv1” (green hypocotyl) was used for gene mapping of the *VrP* gene. The F_2_ population together with their parents was planted in growth chambers with a 14/10 h photoperiod, 250 μE m^–2^ s^–1^ light intensity, 28°C daytime temperature, and 25°C nighttime temperature. A set of 124 mungbean accessions (Supplementary Table 1) was used to investigate the association between *VrP* sequence variations and hypocotyl colors.

### Anthocyanin quantification

An amount of 50 mg of different mungbean or *Arabidopsis* tissue samples were ground into powder in liquid nitrogen, followed by soaking in 1 ml of 1% (v/v) HCl/methanol in the dark for 12 h. The mixture solution was centrifuged at 12,000 × *g* for 10 min. The supernatant was measured for light absorption at 530 and 657 nm by using an ultraviolet spectrophotometer UV-7500 (Shimadzu, Kyoto, Japan). The total anthocyanin content was calculated as described previously (Weiss and Halevy, 1989). The quantification was replicated five times. In the mungbean, the quantification was done for only Sulv1, V2709, and Guilv1.

### Tissue sectioning

Sections of 1 cm of the hypocotyl of a 7-day-old seedling of V2709 and Sulv1 were obtained and embedded in 3% agarose. The sections were made using a double-edge razor blade by hand. Ten sections were placed on a slide with dH_2_O. Images were observed and taken under Olympus BX51 light microscope (Olympus, Tokyo, Japan).

### Mapping of the *VrP* locus

The seedlings of the F_2_ mapping population were recorded for hypocotyl color (purple or green) 2 weeks after sowing, and genomic DNA was extracted from each F_2_ progeny. DNA extraction, primer design, and DNA marker genotyping were the same as described in our previous study (Lin et al., 2020). Briefly, the DNA was extracted using a modified CTAB method. Primers for simple sequence repeats (SSRs) and Insertions/Deletions (InDels; Supplementary Table 2) were designed from all the chromosomes of the mungbean reference sequence “VC1973A” (Kang et al., 2014). In the case of the InDel markers, primers were developed by comparing sequences of Sulv1 (Yan et al., 2020) with the reference sequence. The SSR and InDel markers were screened for polymorphism between the parents. The polymerase chain reaction (PCR) contained 20 ng of DNA template, 10 mM Tris–HCl, 50 mM KCl, 1.5 mM MgCl_2_, 50 mM dNTP, 0.2 mM mixed primers, and 0.5 U *Taq* DNA polymerase. The PCR program consisted of a denaturation step (94°C/5 min), followed by 33 cycles of 94°C/30 s, 55°C/30 s, and 72°C/60 s, and a final extension step of 72°C/5 min. The PCR products were electrophoresed using 8% polyacrylamide gels and DNA bands were visualized by silver staining.

In this study, the green hypocotyl was a recessive trait, thus we considered it the mutant type, while the purple hypocotyl was considered the wild type, thus the hypocotyl color was used as a phenotypic marker. In the F_2_ population, the phenotypic marker, together with 17 molecular markers, including 8 SSR markers and 9 InDel markers (Supplementary Table 2), was used to construct linkage map. The linkage map was constructed using the QTL IciMapping 4.2 software (Meng et al., 2015). A logarithm of odds (LOD) value of 3.0 was used for grouping the markers. Markers were ordered using a recombination counting and ordering algorithm (RECORD) function (van Os et al., 2005). The genetic map distance was calculated using Kosambi’s mapping function (Kosambi, 1944).

### Fine mapping of the *VrP* locus and candidate gene identification

To finely map the *VrP* locus, we initially identified markers associated with this locus by analyzing 10 F_2_ mutant-type (green hypocotyl) individuals using the markers showing polymorphism between their parents (Supplementary Table 2). Markers revealing the same homozygous genotype between all the 10 individuals and their green-hypocotyl parents indicated linkage between the markers and the *VrP* locus. All the markers identifying linkage with the *VrP* locus were used to genotype the whole F_2_ population. Then, the markers were mapped to the mungbean reference genome sequence (Kang et al., 2014), and the location of *VrP* was identified by examining marker recombination events in the F_2_ population.

Genes located between markers flanking the *VrP* locus were identified as candidate genes using the mungbean reference genome sequence annotated by the National Center for Biotechnology Information (NCBI Vigna radiata Annotation Release 101)^1^.

### Phylogenetic analysis

We determined the phylogeny of VrMYB90. Protein sequences from azuki bean [*Vigna angularis* (Ohwi) Ohwi and Ohashi], cowpea [*Vigna unguiculata* (L.) Walp.], soybean (*Glycine max* L. Merr.), petunia (*Petunia bajeensis* T. Ando and Hashim), barrel medic (*Medicago truncatula* Gaertn.), lupin (*Lupinus angustifolius* L.), kiwifruit (*Actinidia chinensis* A. Chev), apple (*Malus* × *domestica* Borkh.), and *Arabidopsis*, matching that of VrMYB90, were identified using the BLASTP algorithm and were downloaded from GenBank. A rooted phylogenetic tree was constructed from the sequences using the neighbor-joining method using the MEGA 10.0 software (Kumar et al., 2018). The robustness of the phylogeny was tested by bootstrapping (1,000 replicates) with the DNAMAN5.2 software (Lynnon Biosoft Corp., Quebec City, QC, Canada).

### Gene sequencing

Eight open-reading frames (ORFs)/genes were identified in a genomic region covering the *VrP* locus (Supplementary Table 3). Coding sequences (CDS) of the eight ORFs in V2709 and Sulv1 were sequenced. In addition, the full ORF of the *VrMYB90* and its 2.56-kb promoter region in V2709, Sulv1, Guilv1 (purple hypocotyl), and KPS2 (green hypocotyl) were sequenced. PCR amplification and Sanger sequencing were performed as per the methods described by Yun et al. (2002). In brief, PCR was conducted using KOD-plus DNA polymerase (Toyobo, Shanghai, China). DNA bands with the expected size were purified using (E.Z.N.A.)^®^ Gel Extraction Kit (Omega Bio-tek, Norcross, GA, United States) and then sequenced using an ABI 3730xl DNA Analyzer (Applied Biosystems, CA, United States) by TSINGKE Biotechnology Co., Ltd. (Nanjing, China).

### Plasmid construction

The coding region without the termination codon of *VrMYB90* from Guilv1 was amplified by polymerase chain reaction (PCR) and recombined into the vector *pCAMBIA1305.1-GFP*. The constructed *35S::VrMYB90-GFP* was used in the following experiments.

### Transient expression in *Nicotiana benthamiana* leaf

*Agrobacterium tumefaciens* strain EHA105 containing the construct *35S::VrMYB90* was infiltrated into the 6-week-old tobacco (*Nicotiana benthamiana* L.) leaf for transient transformation. The transient expression assay was performed as previously described (Yoo et al., 2007). To observe the color change, the infiltrated plants were placed in an artificial illumination incubator for 4–5 days and photographed using a camera.

### *Agrobacterium rhizogenes*-mediated hairy-root transformation

Vector *35S::VrMYB90* was introduced into the *Agrobacterium rhizogenes* K599 strain. Notably, 1-week-old seedlings of Guilv1 were used as receptors. The hairy root transformation was performed as previously described (Kereszt et al., 2007).

### *Agrobacterium tumefaciens*-mediated transformation of *Arabidopsis*

The plasmid was transformed into the *Agrobacterium tumefaciens* EHA105 strain, and an *A. thaliana* transformation was performed using the floral dipping method as described previously (Clough and Bent, 1998). The seeds of transgenic plants were selected on 1/2 Murashige and Skoog media containing 30 μg/ml hygromycin.

### Quantitative real-time PCR analysis

Total RNA was extracted using RNAprep Pure Plant Kit (Tiangen, Beijing, China) following the manufacturer’s instructions. The RNAs from hypocotyls of various mungbean varieties were used for expression analysis of *VrMYB90*. The RNAs from different tissues of Guilv1 were used for the expression pattern investigation of *VrMYB90*. The RNAs from hypocotyls of Guilv1 and transgenic *Arabidopsis* lines were used for expression analysis of anthocyanin-related genes by quantitative real-time PCR (qRT-PCR). First-strand cDNA was synthesized using the Goldenstar^®^ RT6 cDNA Synthesis Kit (Tsingke, Beijing, China). *VrActin3* (*LOC106757568*) gene was used as an endogenous control. Primers used for the qRT-PCR are listed in Supplementary Table 2. The qRT-PCR was carried out according to Lin et al. (2020). The 2^–ΔΔ*CT*^ method was used to calculate relative levels of gene expression.

### Statistical analysis

All values and data points presented were means ± standard deviation (SD). All statistically significant difference tests in this study were conducted using an unpaired two-tailed Student’s *t*-test.

## Results

### Microscopic observation and the genetics of hypocotyl color

**FIGURE 1:**
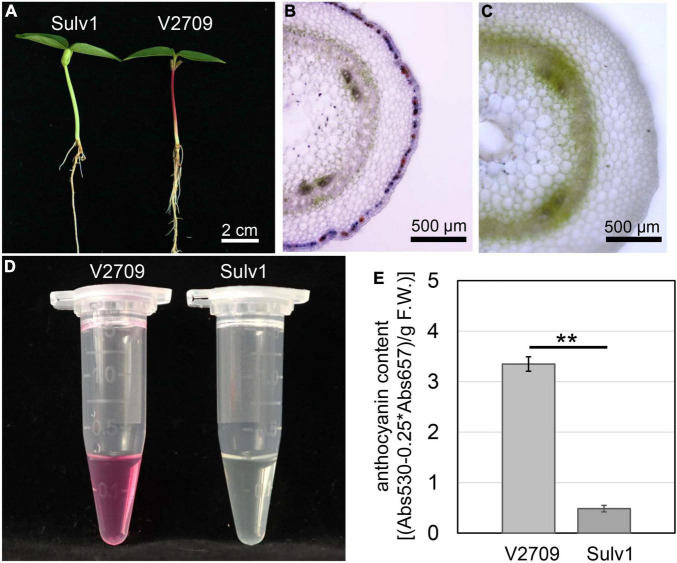
Phenotypes of purple- and green-hypocotyl parental genotypes. **(A)** Green-hypocotyl genotype (Sulv1) and purple-hypocotyl genotype (V2709). **(B)** Transverse section of V2709 hypocotyl. **(C)** Transverse section of Sulv1 hypocotyl. **(D)** Fresh extracts from hypocotyls of V2709 and Sulv1. **(E)** Total anthocyanin contents of V2709 and Sulv1 hypocotyls. **Indicates a significant difference by Student’s *t*-test (*P* < 0.01).

Microscopic observation showed that anthocyanins were accumulated in the second epidermal cell layer that gave rise to a purplish hypocotyl in V2709, and no anthocyanin accumulation was observed in the hypocotyl of Sulv1 (Figures 1A–D). Quantification of anthocyanins revealed that the total anthocyanin contents in the hypocotyl of V2709 were approximately eight times higher than that of Sulv1 (Figure 1E). A color investigation of the hypocotyl in the F_2_ population showed that the segregation ratio of purple individuals to green individuals was consistently 3:1 (χ^2^ = 0.55 < χ*2 0.05* = 3.84, *P* = 0.46). This result was in line with previous studies (Swindell and Poehlman, 1978; Pandey et al., 1989) and indicated that the purple hypocotyl color is a dominant trait controlled by a single locus, designated as *VrP*.

### Mapping of the *VrP* locus and identification of the candidate gene

**FIGURE 2:**
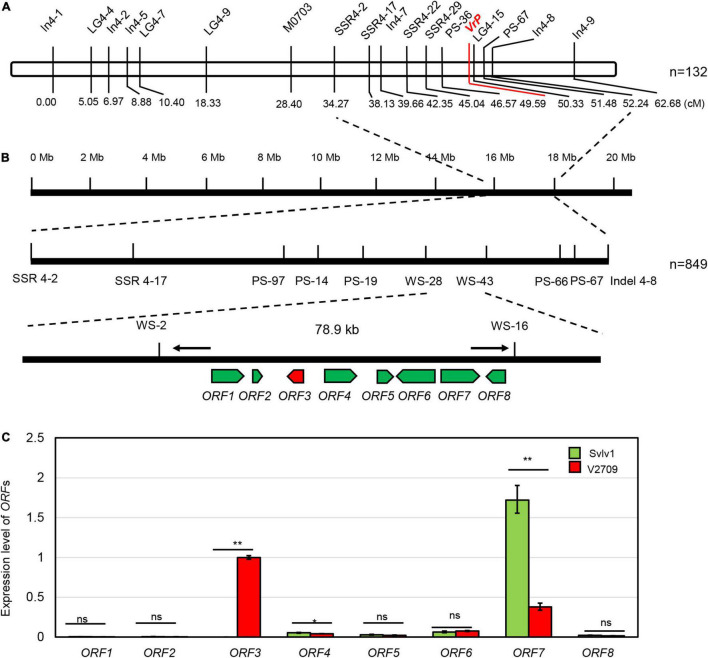
Map-based cloning of *VrP*. **(A)** Linkage map showing the location of *VrP*. The number below indicates the marker position on the linkage map in centimorgan (cM) units, and n indicates the population size for linkage map construction. **(B)** The physical location of the *VrP* gene on chromosome 4 of mungbean. Molecular markers are labeled below the filled bars, arrows represent predicted genes, and n indicates the population size for mapping. **(C)** Expression analysis of ORFs in the mapping region of *VrP* from hypocotyls of V2709 and Sulv1. * and ** indicate a significant difference by Student’s *t*-test at *P* = 0.05 and *P* = 0.01, respectively, while ns indicates a non-significant difference.

Linkage analysis using 132 F2 plants mapped the *VrP* locus to the region between markers PS36 and LG4-15 at a genetic distance of 3.0 and 0.76 cM, respectively (Figure 2A). With all the plants in the population (849 plants) and more markers, the location of the *VrP* locus was precisely mapped between markers WS-28 and WS-43 on chromosome 4 of the mungbean reference genome (Figure 2B).

With more polymorphemic markers explored in the population, the *VrP* locus was narrowed down to a 78.9-kb region, delimited by SSR markers WS-2 and WS-16 (Figure 2B). Based on gene annotation of the mungbean reference sequence by NCBI, there were eight open reading frames (ORFs) predicted in this 78.9-kb region (Figure 2B and Supplementary Table 3). Sequencing and alignment of the CDS of all the eight ORFs between Sulv1 and V2709 revealed polymorphism only in the *ORF3.*

A qRT-PCR experiment showed that *ORF1* and *ORF2* were almost undetectable in both accessions; *ORF5, 6*, and *8* have a slight expression difference in the hypocotyls of Sulv1 and V2709; *ORF7* was expressed significantly higher in Sulv1 than in V2709, while *ORF3* was highly expressed in the hypocotyl of V2709 and undetectable in that of Sulv1 (Figure 2C). According to NCBI annotation, *ORF3* was composed of three exons with a full CDS of 702 bp in length and encoded an R2R3-MYB transcription factor, VrMYB90. The *ORF3* was named “*VrMYB90*.” The *VrMYB90* is highly homologous to *AtMYB90* (*AtPAP2*) in *Arabidopsis* (Zimmermann et al., 2004) and *AcMYB1* in *Actinidia chinensis* Planch (Wang et al., 2019; Supplementary Figure 1B), which forms the MBW complex and promotes the biosynthesis of anthocyanins. Thus, *VrMYB90* was selected as the candidate gene for the *VrP* locus.

**FIGURE 3:**
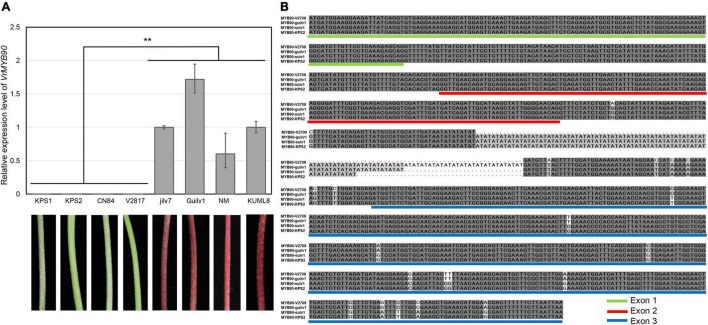
Expression and sequence analysis of *VrMYB90*. **(A)** Expression of *VrMYB90* in eight mungbean accessions; KPS1, CN60, CN84, and V2817 with green hypocotyls Jilv7, V4718, NM, and KUML8 with purple hypocotyls. The expression of *VrMYB90* in Jilv7 was set as control. **(B)** Multiple sequence alignment of VrMYB90 between purple- and green-hypocotyl mungbeans. V2709 and Guilv1 have purple hypocotyls, while Sulv1 and KPS2 have green hypocotyls. Exons are identified by colored underlines. **Indicates a significant difference by Student’s *t-*test (*P* < 0.01).

Since the transcript of *VrMYB90* was almost undetectable in the hypocotyl of Sulv1 (Figure 2C), we then investigated the expression level of *VrMYB90* in four green- and four purple-hypocotyl mungbean cultivars. The expression of *VrMYB90* was nearly nil in all the green-hypocotyl mungbean accessions (KPS1, CN60, CN84, and V2817), but very high in all the accessions with purple hypocotyls (Jilv7, Guilv1, NM10-12, and KUML8) (Figure 3A). These results indicate that the altered expression of *VrMYB90* may be responsible for the variation in the anthocyanin content of hypocotyl in mungbeans.

### Sequence comparison of *VrMYB90*

To explain the difference in the expression of *VrMYB90*, amplified products of the genomic sequence (ATG to TAA) and a 2.56-kb promoter region of *VrMYB90* in four mungbean accessions were sequenced. Sequence alignment revealed numerous SNPs and InDels in the promoter region of the four mungbeans (Supplementary Figure 1A); however, none of those polymorphisms was associated with the phenotype. *VrMYB90* from Sulv1 was the same as the reference sequence (VC1973A; green hypocotyl). *VrMYB90* from KPS2 had the same exonic sequence as Sulv1 with the only difference between them being TA repeats in intron 2 (Figure 3B). Compared with Sulv1, 21 SNPs and a 70-bp deletion existed in *VrMYB90* coding region of V2709, while 13 SNPs and a 42-bp insertion were found in that of Guilv1 (Figure 3B). Among these nucleotide differences, the 13 SNPs in exon 3 were unique in both V2709 and Guilv1. These results suggest that the 13 nucleotide differences in exon 3 of *VrMYB90* are associated with hypocotyl colors in mungbean.

### Functional analysis of *VrMYB90*

**FIGURE 4:**
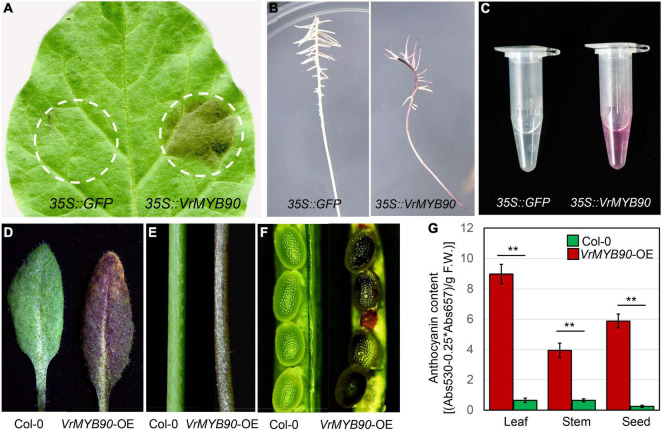
Functional validation of *VrMYB90*. **(A)** Color changes induced by transient expression of *35S::VrMYB90* in *N*. *tabacum* leaves (right) at 5 days after infiltration. *35S::GFP* was used as a negative control (left). Dotted circles indicate infiltrated positions. **(B)** Transient expression of *35S::VrMYB90* induces color changes in mungbean hairy roots. *35S::GFP* was used as a negative control. **(C)** Fresh extracts from the hairy root in **(B)**. **(D–F)** Color changes induced by overexpression of *35S::VrMYB90* in leaf **(D)**, stem **(E)**, and seeds **(F)** of *A. thaliana*. **(G)** The anthocyanin content in different tissues of transgenic *A. thaliana*. **Indicates a significant difference by Student’s *t*-test (*P* < 0.01).

To confirm that *VrMYB90* has a similar function as *AtMYB90* in the regulation of anthocyanin biosynthesis, we overexpressed the CDS of *VrMYB90* from V2709 in different systems. In tobacco leaf, the transient expression of *VrMYB90* led to anthocyanin accumulation in the infection position, while the part injected with an empty *35S::GFP* vector showed no color change (Figure 4A). In the mungbean hairy-root transgenic system, ectopic expression of *VrMYB90* induced anthocyanin accumulation, resulting in a dark purple root, while the mungbean injected by K599 carrying the *35S::GFP* empty vector grew a white root (Figures 4B,C).

After functional verification in transient expression systems, the heterologous expression of *VrMYB90* in *Arabidopsis* was carried out to determine its function in a stable genetic transformation system. The results show that the T_2_-positive transgenic lines exhibited purple leaves, purple stems, and dark brown seeds in the T_2_ generation (Figures 4D–G). These results confirm that *VrMYB90* promotes anthocyanin accumulation in different transgenic plants.

### The correlation Between *VrMYB90* haplotypes and hypocotyl colors in 124 mungbean accessions

**FIGURE 5:**
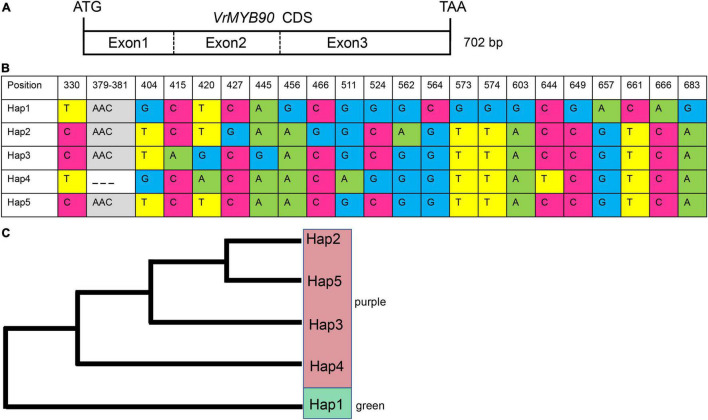
Haplotype analysis of *VrMYB90* in a panel of 124 mungbean accessions. **(A)** Coding sequence (CDS) structure of *VrMYB90*. **(B)** Five haplotypes of *VrMYB90* discovered in 124 mungbean accessions. Nucleotides are labeled with colors and deletions are indicated by dashed lines. **(C)** Phylogenic tree of five haplotypes of *VrMYB90* constructed by the neighbor-joining method.

Our sequencing results of *VrMYB90* from four parental mungbeans suggested that 13 SNPs in exon 3 were associated with the expression levels (Figures 3A,B). To corroborate the correlation between hypocotyl color and *VrMYB90*, we analyzed CDS variations of *VrMYB90* (Figure 5A) in 124 mungbean accessions by Sanger sequencing. Phenotypic analysis of these 124 varieties showed that 40 accessions (32.23%) possessed green hypocotyl and 84 accessions (63.07%) possessed purple hypocotyl (Supplementary Table 1).

The 124 accessions were genotypically classified into five groups of haplotypes based on natural variations, including 21 SNPs and one 3-bp InDel (Figure 5B). All the accessions showing green hypocotyls shared the same haplotype, Hap1, while the accessions displaying purple hypocotyls belonged to either haplotypes Hap2, Hap3, Hap4, or Hap5 (Figure 5C). Among the four haplotypes of purple hypocotyl accessions, Hap3 was the most prevalent haplotype at a frequency of 70.16%, while the haplotypes Hap2, Hap3, and Hap4 were rare, with each haplotype having only one accession. Comparing the Hap1 with other Haps revealed 10 SNPs in exon 3 (positions 456, 564, 573, 574, 603, 649, 657, 661, 666, and 683) associated with the phenotype (Figure 5B). Based on these results, we deduced that these ten SNPs cause the repression of the *VrMYB90* gene.

### Expression analysis of *VrMYB90*

**FIGURE 6:**
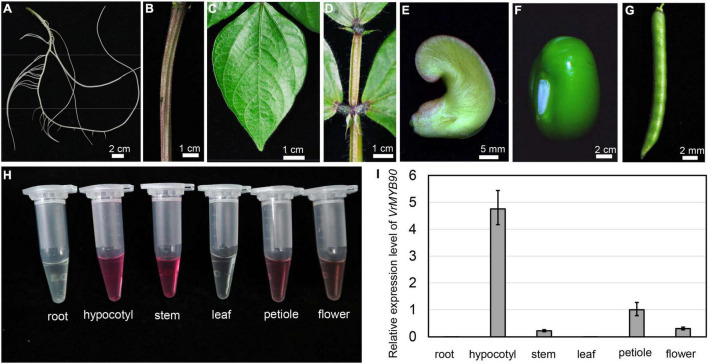
Expression patterns of VrMYB90 in different tissues/parts of mungbean. **(A–G)** Color observation of root **(A)**, stem **(B)**, leaf **(C)**, petiole **(D)**, mature flower **(E)**, young seed **(F)**, and young pod **(G)** of mungbean cultivar Guilv1. **(H)** Fresh extracts from different tissues of Guilv1. **(I)** The expression level of VrMYB90 from different tissues/parts of Guilv1. The expression of *VrMYB90* in petiole was set as one.

Based on color appearance, we noticed differences in anthocyanin accumulation in different mungbean organs. Anthocyanin accumulation was observed in the hypocotyl, stem, petiole, and mature flower, while a few anthocyanins were accumulated in non-purple organs such as the root, leaf, pod, and seed (Figures 6A–G). qRT-PCR analysis revealed that *VrMYB90* showed a great expression level in the hypocotyl and petiole, a low expression level in the stem and flower, and no expression in the leaf and root (Figure 6I). In addition, the expression level appeared to be positively correlated with the anthocyanin content; both the expression level and anthocyanin content were highest in the hypocotyl, followed by the stem and petiole, respectively (Figures 6H,I). Taken together, these results show that *VrMYB90* controls anthocyanin biosynthesis in different organs/tissues, except for the seeds and roots, and the expression level is tightly correlated with a purple color appearance in mungbean.

Pigment accumulation is sensitive to environmental factors, such as light, temperature, and drought, and the anthocyanin content varies greatly depending on the climate and external stresses (Yan et al., 2021). In this study, we observed that the anthocyanin completely disappeared when mungbeans were grown in the dark (Supplementary Figure 2A), and the difference in expression of *VrMYB90* was greatly reduced in the dark (Supplementary Figure 2B). However, when the mungbeans were transferred and exposed to light, *VrMYB90* started to express after 2 h of light exposure, and subsequently, anthocyanin accumulation appeared on the surface of the hypocotyl. The expression level was increased constantly for hours and maintained at a high level (Supplementary Figure 2C). These results show that *VrMYB90* was expressed in several organs of mungbean and was sensitive to light treatment, suggesting that the expression of *VrMYB90* is regulated spatiotemporally.

### Expression analysis of anthocyanin-related genes

**FIGURE 7:**
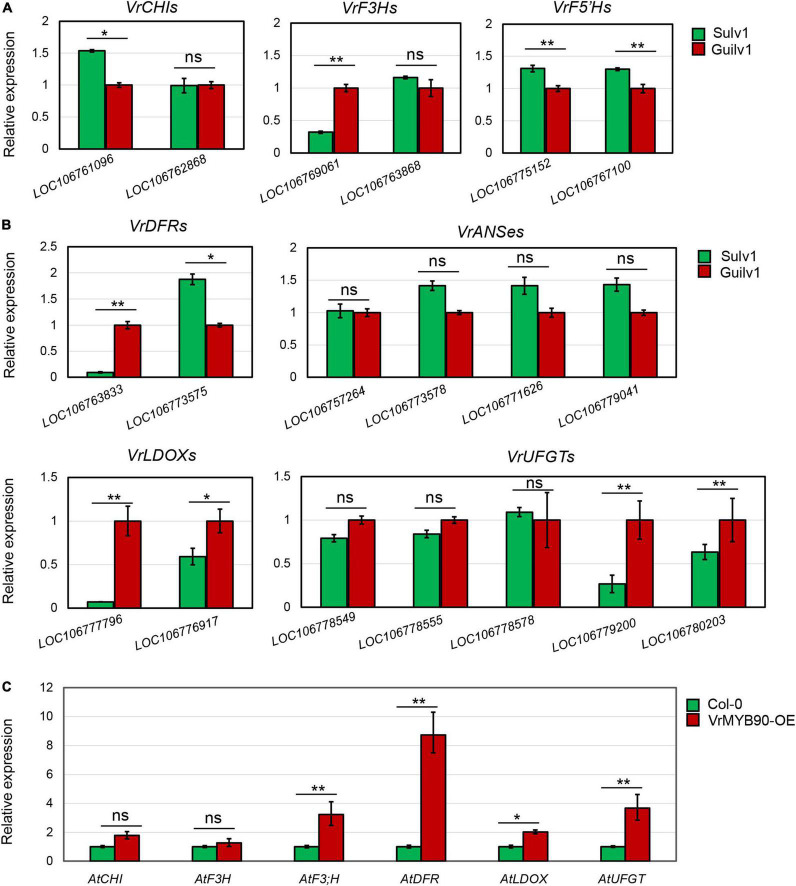
Expression analysis of anthocyanin synthesis genes. **(A,B)** Expression analysis of EBGs **(A)** and LBGs **(B)** in the hypocotyls of Guilv1 and Sulv1. The gene expression in Guilv1 was set as a control. **(C)** Expression analysis of anthocyanin synthesis-related genes in *VrMYB90* over-expression *Arabidopsis thaliana*. The gene expression in wild type (Col-0) was set as a control. * and ** indicate a significant difference by Student’s *t*-test (*P* < 0.05 and *P* < 0.01, respectively).

Expression analysis demonstrated that *VrMYB90* was expressed at a substantially higher level in the hypocotyl than in other organs (Figure 6I). Thus, the RNA samples from the hypocotyl of Guilv1 were prepared for expression analysis of the structural genes. BLAST against the mungbean reference genome (Kang et al., 2014) showed that there are multiple genes encoding different structural genes involved in anthocyanin biosynthesis, including *VrCHI* (*LOC106761096* and *LOC106762868*), *VrF3H* (*LOC106769061* and *LOC106763868*), *VrF5′H* (*LOC106775152* and *LOC106767100*), *VrDFR* (*LOC106763833* and *LOC106773575*), *VrANS* (*LOC106757264, LOC106773578, LOC106771626*, and *LOC106779041*), *VrLDOX* (*LOC106777796* and *LOC106776917*), and *VrUFGT* (*LOC106778549, LOC106778555, LOC106778578, LOC106779200*, and *LOC106780203*). The expression analysis of these genes demonstrated that most of the genes had no significant difference in expression, while an *F3′H* gene (*LOC106769061*), a *DFR* gene (*LOC106763833*), two *LDOX* genes (*LOC106777796* and *LOC106776917*), and a *UFGT* gene (*LOC106779200*) showed significantly higher expression in Guilv1 than in Sulv1 (Figures 7A,B). *F3′H* belongs to the EBG group, while *DFR, LDOX*, and *UFGT* belong to the LBG group. Among these genes, *VrDFR-1* (*LOC106763833*) and *VrLDOX-1* (*LOC106777796*) had a notable expression difference. DFR catalyzes the conversion of dihydroflavonols to leucoanthocyanidins (Meng et al., 2021), and LDOX catalyzes the conversion of leucoanthocyanidins into anthocyanidins (Abrahams et al., 2003), both of which are crucial steps in the biosynthesis of anthocyanins. Additionally, the expression of structural genes in transgenic *Arabidopsis* leaves was measured. The results show that all the structural genes were upregulated in T_3_-positive *VrMYB90* overexpression lines, compared with wild-type *Arabidopsis*, and *AtDFR* exhibited the largest expression difference (Figure 7C). These results indicate that VrMYB90 prefers to promote LBG genes in anthocyanin biosynthesis and that *VrDFR-1* is the most probable target gene of VrMYB90.

## Discussion

### Determinant factors of anthocyanin biosynthesis in mungbean

Pigmentation in mungbean caused by anthocyanin accumulation was observed on the hypocotyls, stem, leaf rachis, petiole, and peduncle. Although the genetics of the coloration of the mungbean hypocotyls is simple, governed by a single dominant locus, *VrP*, and was reported more than six decades ago (Sen and Ghosh, 1959), the molecular basis of the gene underlying the *VrP* locus has never been identified and investigated. The main reason for this is the lack of reference genome information for mungbean. In this study, we exploited the draft genome sequence of mungbean and successfully identified the gene underlying the *VrP* locus.

In this study, we used an F_2_ population to map *VrP* to a 78.9-kb region on chromosome 4 (Figures 2A,B), and the gene *VrMYB90* was isolated and determined to be the factor for anthocyanin accumulation in mungbean hypocotyl. VrMYB90 has high homology with AtMYB90 (PAP2) and AtMYB113 in amino acid sequences (Supplementary Figure 1B) that influenced the color of *Arabidopsis* (Borevitz et al., 2000; Zimmermann et al., 2004). In Sulv1 and other mungbean accessions with green hypocotyls, the transcript of *VrMYB90* was almost abolished, which blocked the biosynthesis of anthocyanins (Figures 2C, 3A). The introduction of *VrMYB90* into different plant systems, e.g., tobacco leaf, mungbean hairy root, and *Arabidopsis*, triggered the expression of structural genes in the anthocyanin synthesis pathway and promoted purple pigment accumulation (Figure 4). qPCR analysis indicated that VrMYB90 upregulates the expression of anthocyanin biosynthesis genes in *Arabidopsis*-positive lines (Figure 7C), which implies it has a similar role to AtMYB90. Moreover, our results indicated that VrMYB90 predominated in the anthocyanin metabolism pathway of mungbean, and the content of anthocyanin positively correlates with the expression level of *VrMYB90* (Figure 6). Together, this study reveals that *VrMYB90* on chromosome 4 is the determiner of anthocyanin synthesis in mungbean.

### Ten single nucleotide polymorphisms in the coding sequences of *VrMYB90* May cause pigment loss

Various nucleotide differences in MYB genes lead to function loss or expression silence and cause a lessened or complete absence of anthocyanin accumulation. A gypsy-like retrotransposon insertion in the promoter of *Vvmyba1* inhibits the expression and turns off anthocyanin synthesis in fruit peels (Kobayashi et al., 2004). A 4-bp insertion in the first exon of *RsMYB1* resulting in a frameshift mutation and premature termination of translation produced white radish cultivars (Kim et al., 2021). A 3.6–3.7 kb fragment insertion in the first intron of *BrMYB2* caused the white-head trait of Chinese cabbage (*Brassica rapa* L.) (He et al., 2020). In this study, sequencing of a 2.6-kb promoter of eight mungbean lines showed several differences; however, none of these uniquely belonged to purple-hypocotyl lines, and thus we believe that the variations in the promoter were not correlated with the expression change of *VrMYB90*. Meanwhile, sequencing the CDS of *VrMYB90* revealed that ten SNPs in exon 3 were tightly related to phenotype (Figure 5). The haplotype analysis revealed that the ten SNPs existed in all green-hypocotyl mungbean accessions (Supplementary Table 1) and further supported that the nucleotide variations in exon 3 caused the disappearance of the *VrMYB90* transcript. Mutations of exons may happen at the target of microRNA or methylation sites, which can lead to an expression change in genes: a non-synonymous SNP mutation in the third exon of *CsCEN* caused a significantly lower expression in shoot apexes and axillary buds in D226, which resulted in determinate growth (Njogu et al., 2020), and an SNP in the third exon of *OsSPL14* perturbed the repression of *OsmiR156*, resulting in a strong expression of *OsSPL14* in the shoot apex, generated an ideal plant architecture and enhanced grain yield (Jiao et al., 2010). However, the specific transcriptional regulation mechanism of the ten SNPs in *VrMYB90* remains unknown.

### Specific expression pattern of *VrMYB90* in mungbean

The tissue expression specificity of MYB genes is common in different species. For example, *AcMYB123* expressed in the inner pericarp of kiwifruit produced the red-fleshed variety “Hongyang” (Wang et al., 2019), while an R2R3 MYB gene *DEEP PURPLE* was uniquely expressed in the petal of *Petunia exserta* J.R. Stehm. and caused diminished red color (Berardi et al., 2021). In our study, measurement of the *VrMYB90* transcript in different organs showed that this gene has different expression levels in different tissues, leading to different anthocyanin contents. Notably, *VrMYB90* was undetectable in mungbean seeds. Noble et al. (2018) reported that anthocyanins induce the diversity of seed coat colors in mungbean and *VrMYB113*, which is highly homologous to *VrMYB90* and may be a determinant gene controlling seed color. In contrast to this report, the results from our study suggest that the seed coat color of some black seed mungbean accessions may be governed by another MBW complex. This is supported by the limited expression of *VrMYB90* in the seeds and the imperfect correlation between the hypocotyl color and the seed coat color in the 124 mungbean accessions (Supplementary Table 1); for example, Anhei2, a mungbean accession, expressed a green hypocotyl but a black seed coat. Altogether, we believe that a complex regulatory mechanism of anthocyanin production exists in mungbean.

## Data availability statement

The datasets presented in this study can be found in online repositories. The names of the repository/repositories and accession number(s) can be found in the article/Supplementary Material.

## Author contributions

YL, PS, and XC conceived and designed the study and wrote and revised the manuscript. PS supervised the study. YL, KL, JL, JC, and XY performed the gene mapping, sequencing, and transformation. YL conducted the microscopic observation, bioinformatics, and data analyses. XC and PS secured research funds. All authors reviewed and approved the manuscript.

## Conflict of interest

The authors declare that the research was conducted in the absence of any commercial or financial relationships that could be construed as a potential conflict of interest.

## Publisher’s note

All claims expressed in this article are solely those of the authors and do not necessarily represent those of their affiliated organizations, or those of the publisher, the editors and the reviewers. Any product that may be evaluated in this article, or claim that may be made by its manufacturer, is not guaranteed or endorsed by the publisher.

## Footnotes

1
https://www.ncbi.nlm.nih.gov/genome/annotation_euk/Vigna_radiata/101
