# Supplementary material for: The mungbean VrP locus encoding MYB90, an R2R3-type MYB protein, regulates anthocyanin biosynthesis
Source: Front Plant Sci. 2022 Jul 22;13:895634. doi: 10.3389/fpls.2022.895634 (PMC9355716; doi:10.3389/fpls.2022.895634)

Supplementary Figure S2. Expression response to light of *VrMYB90*. (A) Anthocyanins accumulation after light exposure. (B) Expression difference of *VrMYB90* between dark and light treatment. (C) Expression level change of *VrMYB90* at different times after light treatment.

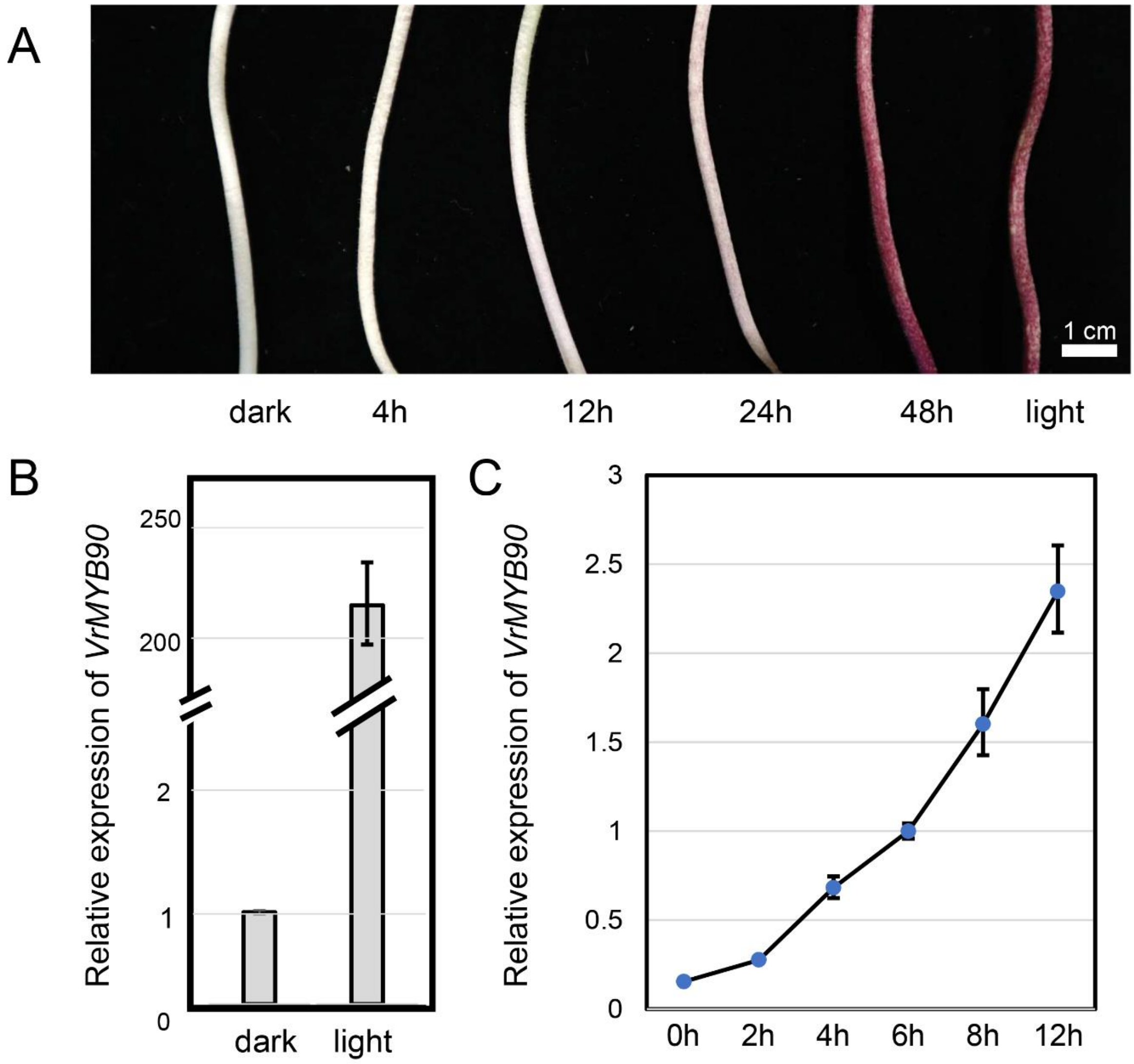

Supplement: Supplementary file 2 [file Data_Sheet_2.PDF]
