# Supplementary material for: The mungbean VrP locus encoding MYB90, an R2R3-type MYB protein, regulates anthocyanin biosynthesis
Source: Front Plant Sci. 2022 Jul 22;13:895634. doi: 10.3389/fpls.2022.895634 (PMC9355716; doi:10.3389/fpls.2022.895634)

Supplementary Figure S1. Sequence analysis of *VrMYB90* promoter and protein. (A) 2.56-kb promoter sequence alignment of *VrMYB90* from different mungbean accessions. (B) Phylogenetic analysis of VrMYB90 and its homologous proteins.

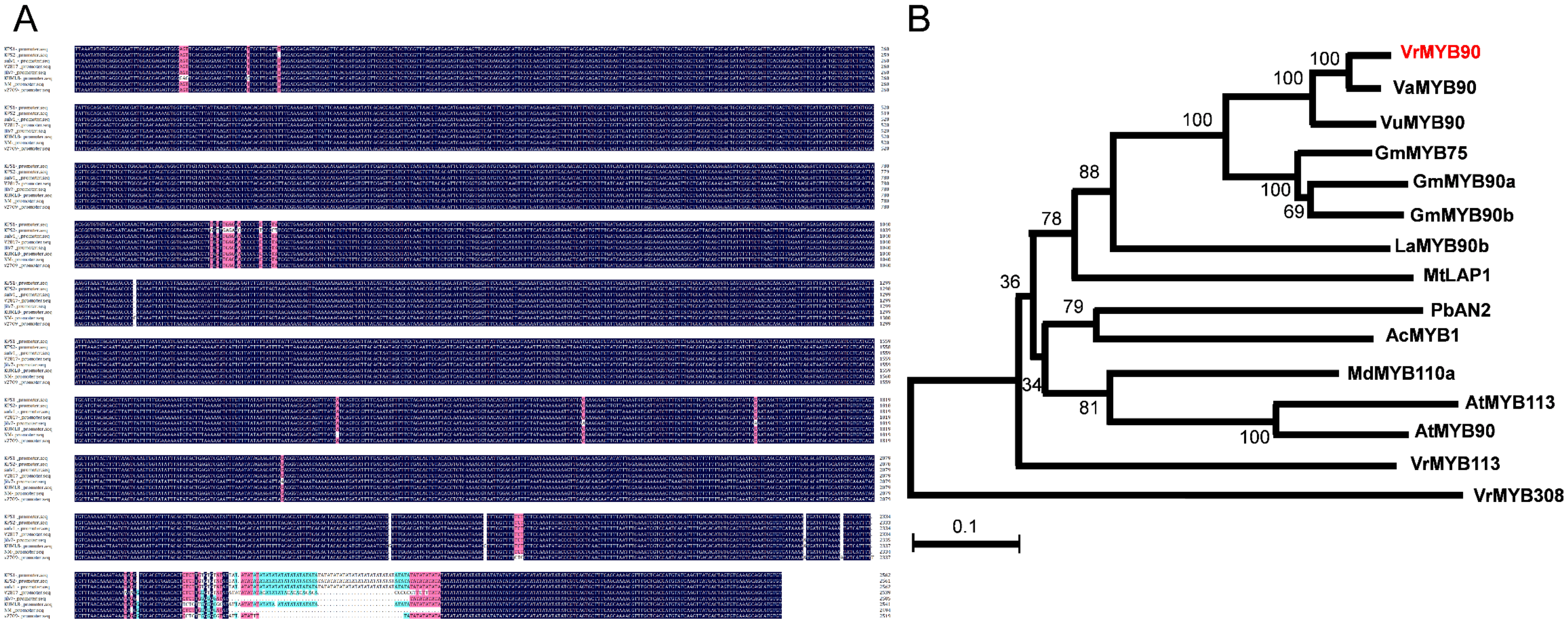

Supplement: Supplementary file 1 [file Data_Sheet_1.PDF]
